# Single-plane retroperitoneoscopic adrenalectomy: a new operative procedure for benign adrenal disease

**DOI:** 10.1038/s41598-018-22433-3

**Published:** 2018-03-05

**Authors:** Songchao Li, Jun Wang, Erwei Zhang, Wansheng Gao, Jinjian Yang, Zhankui Jia

**Affiliations:** 1 Department of Urology, The First Affiliated Hospital of Zhengzhou University, Jianshe Road, Erqi District, Zhengzhou City, 450052 Henan Province People’s Republic of China; 2 Urology Laboratory, The First Affiliated Hospital of Zhengzhou University, Urological Institute of Henan, Jianshe Road, Erqi District, Zhengzhou City, 450052 Henan Province People’s Republic of China; 3 Tumor Molecular Biology Key Laboratory of Zhengzhou, The First Affiliated Hospital of Zhengzhou University, Jianshe Road, Erqi District, Zhengzhou City, 450052 Henan Province People’s Republic of China

## Abstract

To evaluate the therapeutic effect of single-plane retroperitoneoscopic adrenalectomy. From February 2014 to March 2017, 251 patients underwent single-plane retroperitoneoscopic adrenalectomy, and their operative outcomes were compared with those of 98 patients who underwent anatomical three-plane retroperitoneoscopic adrenalectomy. Among 35 patients with a body mass index (BMI) of ≥30 kg/m^2^, their operative outcomes were compared between two operative procedures. The demographic data and perioperative outcomes of the patients were statistically analysed. The single-plane and three-plane groups were comparable in terms of estimated blood loss, time to oral intake, hospital stay, and incidence of complications among patients with similar baseline demographics. The single-plane group had a significantly shorter operation time (46.9 ± 5.8 vs 54.8 ± 7.0 mins, P < 0.0001) and lower analgesia requirement (56/251 vs 33/98, p = 0.03). For obese patients with a BMI of ≥30 kg/m^2^, single-plane adrenalectomy was also associated with a significantly shorter operation time(48.1 ± 6.2 vs 64.1 ± 5.1 mins, p < 0.0001). Single-plane retroperitoneoscopic adrenalectomy is feasible, safe, and effective in the treatment of adrenal masses <5 cm in size and provides a shorter operation time and better pain control than anatomical retroperitoneal adrenalectomy, especially in obese patients.

## Introduction

Minimally invasive adrenalectomy has been the gold standard treatment for benign adrenal tumours for the past two decades. Laparoscopic transperitoneal adrenalectomy is the most widely practiced technique because it offers a clear anatomical view and large working space. However, with improvements of laparoscopic instruments and surgical techniques, posterior retroperitoneal adrenalectomy is now regarded as a better approach for small benign adrenal tumours in terms of a shorter operative time, reduced blood loss, reduced length of hospital stay, less postoperative pain, and faster recovery^1,2^.

In retroperitoneoscopic adrenalectomy, access to the adrenal gland routinely proceeds from initial dissection of the renal hilum and renal vein to resultant identification of the adrenal vein and then to the adrenal gland^3^. Another adrenalectomy technique involves initial dissection of the perinephric fat covering the kidney and adrenal gland from the surrounding muscles. The adrenal vein is finally reached, clipped, and divided^4,5^. Retroperitoneal adrenalectomy can be performed through a dorsal approach^6^. In these series, however, the mean operative time was 65 to 194 minutes and the complication rate was 3.2% to 11.0%^3–6^. Anatomic retroperitoneal adrenalectomy, which is characterised by “three bloodless planes” through a lateral retroperitoneal approach, has been proven safe, effective, and technically efficient for surgical treatment of adrenal disease. Its mean operative time in previous studies was 45.0 ± 19.1 mins, and its complication rate was 1.5%^7,8^. We performed three-plane anatomic retroperitoneal adrenalectomy in our institution before 2015. However, we found we can perform adrenalectomy by only dissecting the ventral side of the kidney, without dissection of the lateral side and upper pole. We call this procedure single-plane adrenalectomy. The purpose of this study was to compare the perioperative outcomes of single-plane versus three-plane retroperitoneoscopic adrenalectomy.

## Results

The baseline demographic data were broadly comparable between the two groups with respect to patient age, sex, BMI, tumour size, and tumour laterality (Table 1). Surgery was performed in all 251 patients undergoing single-plane retroperitoneoscopic adrenalectomy. Anatomical retroperitoneal adrenalectomy was successful in all 98 patients. Conversion to open surgery was not necessary in any case. One patient with an adrenal tumour adjacent to the renal hilum was converted to anatomical adrenalectomy.

**Table 1:** Patient demographics.

	Single plane group	Three planes group	P
No. of patients	251	98	
Age (years)	47.7 ± 5.9	46.2 ± 4.5	0.38
Sex (M/F), n	119/132	45/53	0.8
BMI (kg/m^2^)	26.6 ± 4.6	27.3 ± 4.7	0.58
Tumor size, cm	2.1 ± 0.51	2.4 ± 0.54	0.2
Tumor side (right/left), n	131/120	48/50	0.59
Clinical diagnosis			
Primary aldosteronism	106	40	0.81
Cushing’s syndrome	37	18	0.4
Pheochromocytoma	55	25	0.47
Nonfunctional tumor	53	15	0.22

Data are presented as n or mean ± standard deviation. M, male; F, female; BMI, body mass index.

Table 2 summarises the intraoperative outcomes. Compared with the three-plane group, the single-plane group had a significantly shorter operation time (46.9 ± 5.8 vs. 54.8 ± 7.0 mins, respectively; p < 0.0001) and lower in-hospital analgesic requirement (56/251 vs 33/98, p < 0.03). The mean patient’s visual analogue pain scale (VAPS) score (0–10, with 10 corresponding to the most intense pain) at 24 hours was 4.5 ± 1.7 in the single-plane group and 5.8 ± 1.7 in the three-plane group (p = 0.02). The patients from both groups displayed no significant differences in estimated blood loss, time to resumption of oral intake, VAPS score at discharge, length of hospital stay, or incidence of complications.

**Table 2:** All patients, perioperative outcomes.

	Single plane group	Three planes group	P
No. of patients	251	98	
Operative time(mins)	46.9 ± 5.8	54.8 ± 7.0	P < 0.0001
EBL(mL)	24.5 ± 9.2	25.1 ± 7.0	P = 0.63
No. of complications	15	5	P = 0.75
Peritoneum tear	5	2	
Subcutaneous emphysema	8	2	
Postoperative fever	2	1	
Wound infection	0	0	
Analgesia requirement(N)	56	33	P = 0.03
VAPS at 24 h	4.5 ± 1.7	5.8 ± 1.7	P = 0.02
VAPS at discharge	1.5 ± 0.8	1.7 ± 0.8	P = 0.52
Oral Intake(hours)	23.6 ± 3.0	24.4 ± 3.3	P = 0.46
Hospital stay(d)	6.8 ± 0.9	7.2 ± 0.9	P = 0.34

Data are presented as n or mean ± standard deviation.

EBL, estimated blood loss; VAPS, visual analogue pain scale.

In the single-plane group, five patients developed intraoperative peritoneal tears, eight developed postoperative subcutaneous emphysema (a grade I complication), and two developed postoperative pyrexia (a grade II complication). In the three-plane group, two patients developed intraoperative peritoneal tears, two developed postoperative subcutaneous emphysema, and one developed postoperative pyrexia. All complications were treated conservatively. During a mean follow-up of 22.1 months (range, 6–43 months), no tumour recurrence or death was documented in either group; however, three patients were lost to follow-up.

Among the 35 patients with obesity (BMI ≥30 kg/m^2^), 22 underwent single-plane adrenalectomy and 13 underwent anatomical retroperitoneal adrenalectomy. The baseline demographic data were broadly comparable between the two groups (Table 3). We also compared the intraoperative outcomes of the two groups (Table 4). Compared with the anatomical three-plane group, the single-plane group had a significantly shorter operation time (48.1 ± 6.2 vs. 64.1 ± 5.1 mins, p < 0.0001) and broadly comparable complication rate (9.1% vs. 7.6%, p = 0.89).

**Table 3:** Demographics of patients with a body mass index of ≥30 kg/m^2^.

	Single plane group	Three planes group	P
No. of patients	22	13	
Age (years)	46.1 ± 3.8	41.2 ± 3.9	0.22
Sex (M/F), n	10/12	5/8	0.69
BMI (kg/m^2^)	32.4 ± 2.3	33.0 ± 2.2	0.55
Tumor size, cm	2.4 ± 0.4	2.2 ± 0.54	0.45
Tumor side (right/left), n	9/13	6/7	0.76
Clinical diagnosis			
Primary aldosteronism	10	6	0.97
Cushing’s syndrome	3	1	0.59
Pheochromocytoma	4	4	0.39
Nonfunctional tumor	5	2	0.6

Data are presented as n or mean ± standard deviation.M, male; F, female; BMI, body mass index.

**Table 4:** Perioperative outcomes of patients with a body mass index of ≥30 kg/m^2^.

	Single plane group	Three planes group	P
No. of patients	22	13	
Operative time (mins)	48.1 ± 6.2	64.1 ± 5.1	P < 0.0001
EBL(mL)	23.3 ± 7.1	27.3 ± 3.3	P = 0.06
No. of complications	2	1	P = 0.89
Peritoneum tear	1	0	
Subcutaneous emphysema	1	0	
Postoperative fever	0	1	
Wound infection	0	0	
Analgesia requirement(N)	9 4	P = 0.55	
VAPS at 24 h	5.6 ± 1.8	5.4 ± 1.8	P = 0.7
VAPS at discharge	1.6 ± 0.7	1.8 ± 0.7	P = 0.43
Oral Intake(hours)	25 ± 3.9	24 ± 3.6	P = 0.5
Hospital stay(d)	6.9 ± 0.8	7.1 ± 0.5	P = 0.44

Data are presented as n or mean ± standard deviation. EBL, estimated blood loss; VAPS, visual analogue pain scale.

## Discussion

Over the past several decades, urologists have been attempting to improve the safety and reduce the invasiveness of surgery by exploring or modifying new surgical approaches. Many studies have focused on laparoendoscopic single-site, natural orifice transluminal endoscopic surgery, and robotic surgery. A deeper understanding of these processes has revealed their disadvantages, including long operation times, instrument collision, difficult operations, and the need for several robot arms/trocars. Compared with the established treatment options, any novel alternatives must be superior (or at least equivalent) in terms of safety, efficacy, and treatment outcomes^9^.

Anatomic retroperitoneoscopic adrenalectomy, first reported by Zhang *et al*. in 2007, greatly shortened the operation time and decreased the complication rate. We adopted anatomical three-plane retroperitoneal adrenalectomy before 2015. However, we found that this surgical method has disadvantages. The upper pole of the kidney is completely dissected after three-plane dissection. Additionally, nephroptosis is a risk in slim patients, and the procedure is time-consuming in patients with perinephric adhesion due to diabetes or a surgical history. To overcome these disadvantages, we modified anatomic retroperitoneal adrenalectomy and performed adrenalectomy through only dissection of the ventral side of the kidney. This surgical approach is relatively simple and results in little damage to the upper pole of the kidney. The present study demonstrated that compared with anatomical three-plane adrenalectomy, single-plane adrenalectomy had a significantly shorter operation time and lower analgesic requirement for adrenal tumours of <5 cm. This may due to two reasons. First, single-plane adrenalectomy does not require excessive isolation of the lateral side and upper pole of the kidney. Second, single-plane adrenalectomy does not increase the difficulty of the operation.

The prevalence of overweight and obese patients has been increasing at a rapid rate and is currently a global problem^10,11^. The growing population of obese patients seems to bring more technical challenges to surgeons. The high BMI of patients with obesity may be associated with operative complexity and an additional risk of complications^12,13^. The maturity of surgical techniques has brought about an emerging controversy regarding whether obesity increases complications in laparoscopy. Obesity has been found to be associated with a prolonged operation time and does not directly increase perioperative complications^14,15^. In the current study, we compared perioperative outcomes between single-plane adrenalectomy and anatomical three-plane adrenalectomy for patients with a BMI of ≥30 kg/m^2^. The results showed that for obese patients, single-plane adrenalectomy also had a significantly shorter operation time and did not increase perioperative complications compared with anatomical three-plane adrenalectomy. Moreover, the operation time of single-plane adrenalectomy was comparable between obese patients and the total patient population. This result was mostly due to the main advantage of single-plane adrenalectomy; namely, that it does not require isolation of the lateral side of the kidney and removal of excessive perirenal fat, which is time-consuming, especially in obese patients. Difficulties caused by obesity during single-plane adrenalectomy, such as a limited operative space or difficulty in adrenal identification, did not bring more technical challenges to surgeons. Additionally, adhesion within the operation field increases complications and the operative time^16^. For patients with perinephric adhesion due to diabetes or a surgical history, anatomical three-plane adrenalectomy may be more difficult and time-consuming because of excessive isolation of the lateral side and upper pole of the kidney. The superiority of single-plane adrenalectomy may be more obvious in these patients. We are doing to verify that in following study.

Investigations of the limits of the retroperitoneal approach suggest that the procedure is ideally performed for masses of <5 to 7 cm^17,18^. However, Hwang *et al*.^5^ considered that retroperitoneal laparoscopic adrenalectomy can be used in patients with tumours of >5 cm, and Wang *et al*.^19^ reported that anatomic retroperitoneoscopic adrenalectomy is a safe and feasible procedure for large adrenal masses with a diameter of >7 cm. However, this procedure results in a longer operation time and greater blood loss. Considering the smaller operative space provided by single-plane adrenalectomy compared with anatomical adrenalectomy, we excluded patients with adrenal tumours of >5 cm. We found that the smaller operative space did not affect experienced surgeon’s performance of single-plane adrenalectomy for adrenal tumours of <5 cm. Only when dealing with a branch of the inferior renal arteries do we often need to press the kidney down and outward by the fourth trocar to increase the operative space. Only one patient with an adrenal tumour adjacent to the renal hilum was converted to anatomical adrenalectomy to better expose the renal hilum. All operations in this study were performed by the same experienced surgeon. However, unskilled young surgeons can perform single-plane adrenalectomy smoothly in our routine work. This reflects the fact that single-plane adrenalectomy is not difficult.

Because this study included only one surgeon, the results might depend largely on different urologists’ personal habits. Further large-scale prospective studies of single-plane retroperitoneoscopic adrenalectomy are warranted to demonstrate its safety and advantages over anatomical three-plane adrenalectomy.

## Conclusion

Single-plane retroperitoneoscopic adrenalectomy is feasible, safe, and effective in the treatment of adrenal masses <5 cm in size and provides a shorter operation time and better pain control than traditional anatomical retroperitoneal adrenalectomy, especially in obese patients.

## Patients and Methods

### Patients

This retrospective study was approved by ethics committee of the First Affiliated Hospital, Zhengzhou University, Zhengzhou, Henan Province, China. Written informed consent was obtained from all study participants. Patient demographics, surgical details, and perioperative outcomes were collected and assessed, in accordance with relevant guidelines and regulations and supervised by ethics committee of the First Affiliated Hospital, Zhengzhou University. From February 2014 to March 2017, 349 patients with benign adrenal tumours underwent surgical treatment at our institution. Of these, 251 underwent single-plane retroperitoneoscopic adrenalectomy and 98 underwent anatomic retroperitoneoscopic adrenalectomy. Among 35 patients with obesity (BMI ≥30 kg/m^2^), 22 underwent single-plane adrenalectomy and 13 underwent anatomical three-plane retroperitoneoscopic adrenalectomy. All procedures were performed by the same senior surgeon, and in all cases, the diameter of the tumour was <5.0 cm. Patients were excluded from this study if they had bilateral adrenal tumours or a high suspicion of malignancy. All patients were evaluated preoperatively by computed tomography or magnetic resonance abdominal imaging. The clinical characteristics of the patients are shown in Table 1.

Data collected included patient demographics, surgical details, and perioperative outcomes. Postoperative complications were graded using the Clavien–Dindo classification system^20^. VAPS score was recorded every 24 hours after the operation until discharge. Analgesic use from arrival in the post-anaesthesia care unit to discharge was analysed.

### Operative techniques

The well-established conventional retroperitoneal laparoscopic adrenalectomy technique was used^8^. The patient is placed in a 90-degree lateral decubitus position on the operating table which must has kidney bridge, with the pathological adrenal on the upside. An axillary roll is deployed under the patient caudal to the axilla to avoid the potential compression of branchial plexus and another roll is deployed under the patient’s waist. The patient’s lower leg is flexed to 90 degree and separated from the upper leg by a pillow. Then, the patient is secured to the table with straps at hips and knees. Finally, the operating table is flexed to increase the working space between the costal margin and iliac crest.

The procedure was generally performed with three ports. After incision of Gerota’s fascia, three relatively bloodless planes were dissected to expose and separate the adrenal gland. The first dissection plane between the perirenal fat and the anterior renal fascia was located on the superomedial side of the kidney. The second plane between the posterior renal fascia and the lateral aspect of the perirenal fat expanded the operative space. The third plane was located on the parenchymal surface of the upper renal pole. Finally, the adrenal specimen was retrieved through the posterior axillary trocar port site

**Figure 1:**
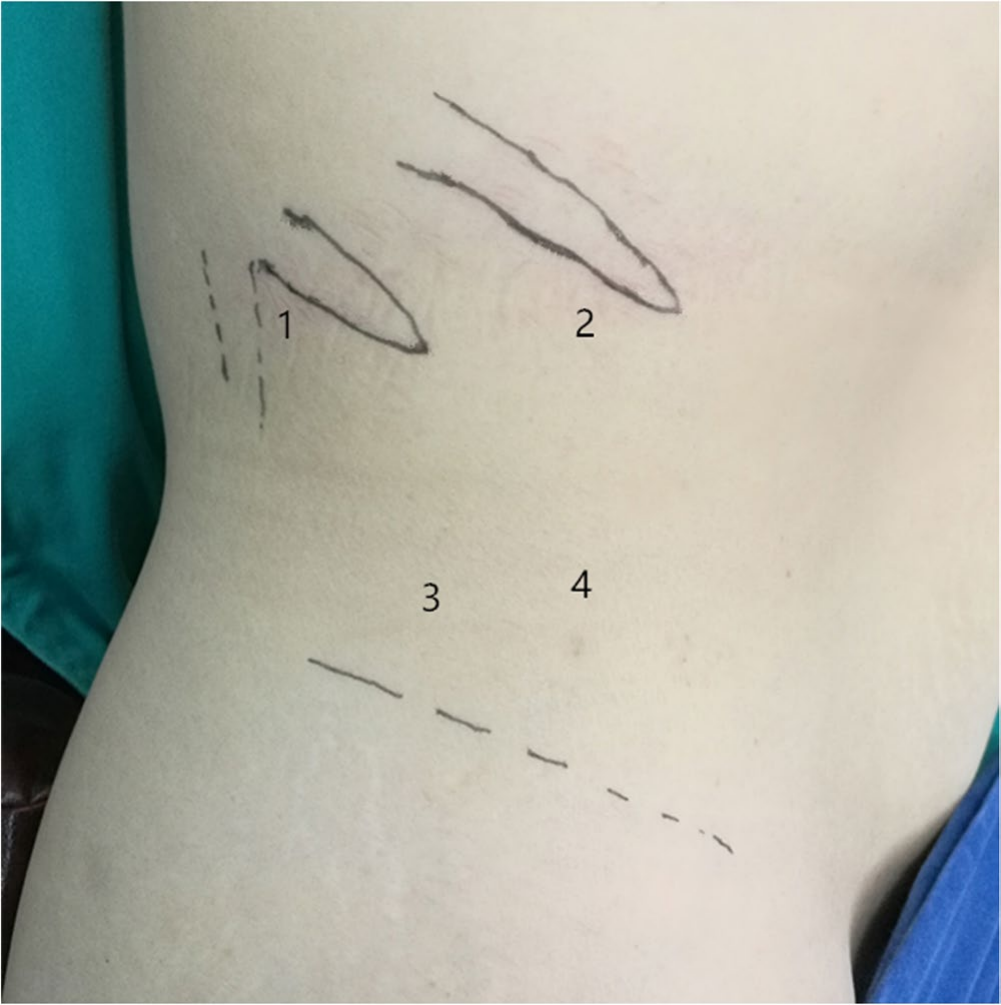
Port sites for single-plane retroperitoneal adrenalectomy. 1, 12-mm port below the 12th rib in the posterior axillary line. 2, 12-mm port (right side) or 5-mm port (left side) under the subcostal margin in the anterior axillary line. 3, 10-mm port above the iliac crest in the midaxillary line for the laparoscope. 4, 5-mm port placed ventral to and along a transverse line with the trocar above the iliac crest.

**Figure 2:**
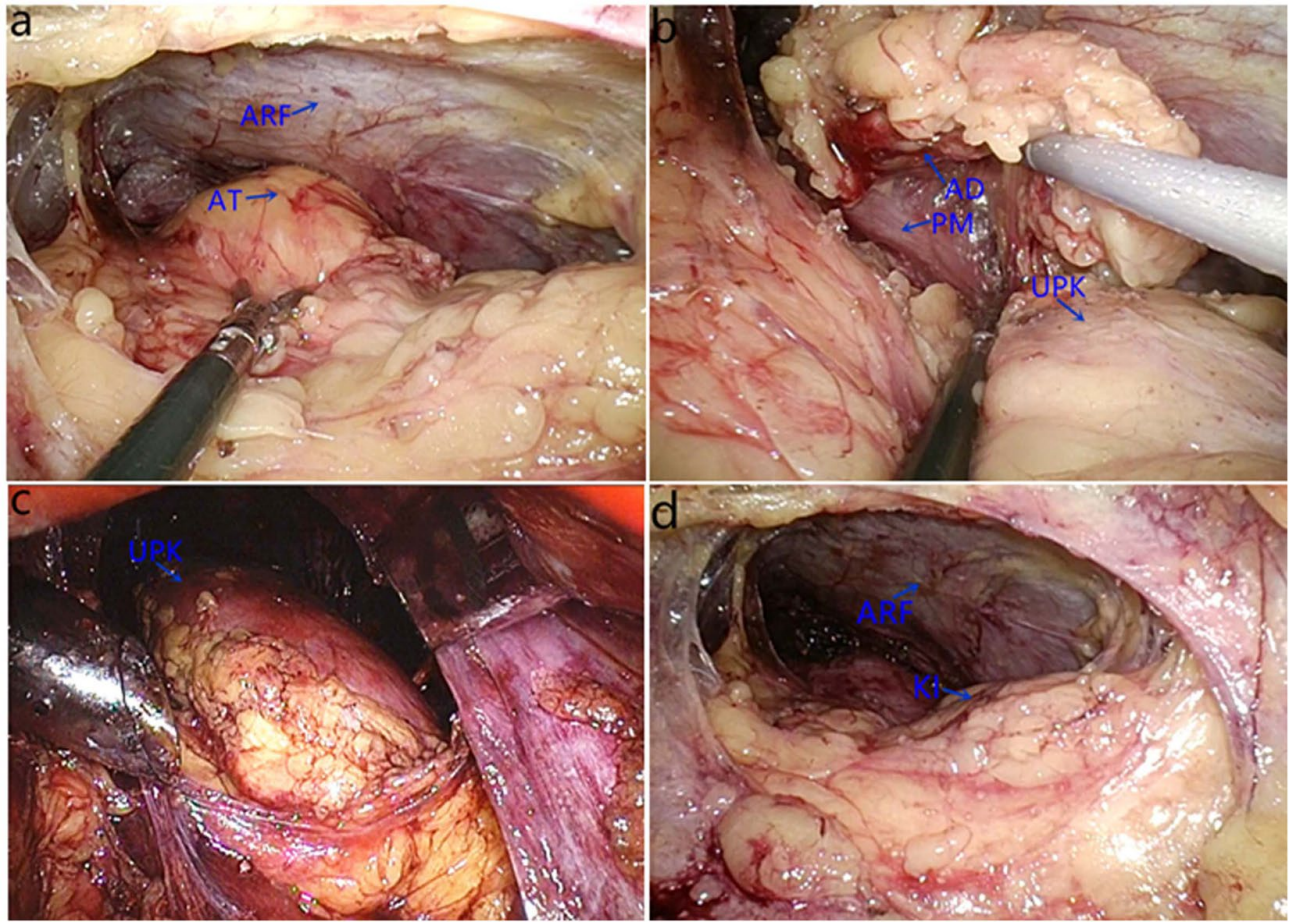
Key procedures of single-plane retroperitoneal adrenalectomy (right side) and major intraoperative anatomical structures. (**a**) The anterior aspect of the adrenal tumour (AT) with perirenal fat was completely separated from anterior renal fascia(ARF). (**b**) The lateral side and bottom of the adrenal gland was exposed after dissection between the adrenal gland (AD) bottom and parenchymal surface of the upper kidney(KI) pole. psoas muscle(PM). (**c**) Anatomical three-plane adrenalectomy involves dissection of ventral side, lateral side and upper pole of kidney(UPK). (**d**) Single-plane adrenalectomy only involves dissection of ventral side of the kidney.

Patient positioning and placement of the first three trocars in patients who underwent single-plane retroperitoneal adrenalectomy was similar to that in patients who underwent traditional three-plane retroperitoneal adrenalectomy. The fourth trocar was placed ventral to and along a transverse line with the trocar above the iliac crest. The distance between the two trocars was about 3 cm (Fig. 1). After clearing the retroperitoneal fat, we dissected the plane of perirenal fat and the anterior renal fascia and completely expose the anterior aspect of adrenal tumor or gland in the first stage of the operation (Fig. 2a). The fat on the lateral border of the adrenal was then lifted, and dissection was performed between the parenchymal surface of the upper renal pole and adipose capsule at the bottom of the adrenal gland to expose the lateral side and bottom of the adrenal gland (Fig. 2b). On the left side, after dividing the branch of the inferior renal arteries, the central vein was completely exposed, clipped with a Hem-o-lock, and transected. On the right side, usually after dividing the inferior adrenal arteries and lifting the inferior margin of the adrenal gland, the central vein could be completely exposed and divided. Finally, the upper adrenal artery and its surrounding fat tissue was transected. Once dissection was completed, the specimen was entrapped in a retrieval bag and extracted through the posterior-axillary trocar port site. If necessary, the skin incision can be suitably prolonged. The fourth trocar was used to press the kidney downward and outward to completely expose the inferior adrenal arteries. We performed either partial or total adrenalectomy depending on the patient’s different condition. Compared with anatomical three-plane adrenalectomy (Fig. 2c), single-plane adrenalectomy involves only dissection of the ventral side of the kidney without dissection of the lateral side and upper pole (Fig. 2d).

### Outcome evaluation

The operation time was defined as the period from skin incision to skin closure. The operation time and blood loss volume were obtained from the surgeon’s operation record or the anaesthesia record.

### Statistical analysis

The data are expressed as mean with standard deviation. Comparisons were performed using the X^2^ test, Student’s t test, and Mann–Whitney U test for categorical and continuous variables. The statistical analysis was performed using SPSS 17.0 (SPSS Inc., Chicago, IL, USA), and p-values of <0.05 were considered statistically significant.

### Data availability statement

The datasets generated during and/or analysed during the current study are available from the corresponding author on reasonable request.
